# Deep learning for contrast-enhanced MRI in pediatric brain imaging

**DOI:** 10.1007/s00234-026-04073-x

**Published:** 2026-07-04

**Authors:** Anna Macula, Giovanni Morana, Fiorenza Coppola, Andrea Garnero, Andrea Rossi, Alberto Fringuello Mingo, Stefano Tambalo, Giovanni Valbusa, Fabio Tedoldi, Sonia Colombo Serra, Angelo Bifone

**Affiliations:** 1 Department of Physics, University of Turin, Turin, Italy; 2 Bracco (Italy), Colleretto Giacosa, Italy; 3 Neuroradiology Unit, Azienda Ospedaliera Citta’ della Salute e della Scienza di Torino, Turin, Italy; 4 Neuroradiology Unit, Istituto Giannina Gaslini, Genoa, Italy; 5 Department of Health Sciences, University of Genoa, Genoa, Italy; 6 Centre for Mind/Brain Sciences, University of Trento, Trento, Italy; 7 Department of Molecular Biotechnology and Health Sciences, University of Turin, Turin, Italy

**Keywords:** Pediatric, Contrast amplification, Brain, MRI, Deep learning

## Abstract

**Purpose:**

A deep learning algorithm for contrast amplification in brain MRI, trained exclusively on adult data, was tested for cross-population generalization to pediatric patients, including subjects aged 0–2 years.

**Methods:**

A retrospective monocentric dataset (n = 22 cases) comprising pediatric patients (0–17 years old) diagnosed with various brain tumors was used to evaluate the algorithm, which takes T1-weighted pre- and standard post-contrast images as input and generates an output image with amplified contrast, further post-processed with an HDR algorithm. Quantitative comparisons between standard and amplified images were performed using contrast-to-noise ratio (CNR), contrast enhancement percentage (CEP), and lesion-to-background ratio (LBR). Three neuroradiologists performed qualitative assessment using a 4-point Likert scale, focusing on lesion contrast and delineation. Anatomical similarity was assessed using SSIM and log-Jacobian range. Statistical significance was evaluated using two-tailed paired t-tests.

**Results:**

Compared to standard-dose images, contrast-amplified images showed significantly higher values for CNR (+ 186.5%), LBR (+ 61.9%), and CEP (+ 110.4%). Qualitative assessments demonstrated comparable lesion visualization, with improvements observed in selected cases. Reader 1 preferred the contrast-amplified image in 12 of 22 cases (54.5%), reader 2 favored it in 18 of 22 cases (81.8%) and reader 3 in 13/22 cases (59.1%). One reader reported improved overall image quality (mean score: 3.95 vs. 3.73). The average SSIM between amplified and standard-dose images was 0.98, and any significant anatomical differences were highlighted by the log-Jacobian range (*p-*value = 0.556).

**Conclusion:**

An algorithm for contrast amplification based on deep learning, trained with adult data, significantly enhances quantitative contrast metrics in images from pediatric patients. It is preferred over standard-dose images in the majority of cases when used for pediatric brain MRI, indicating its promising application for cross-population applicability.

**Supplementary Information:**

The online version contains supplementary material available at https://doi.org/10.1007/s00234-026-04073-x.

## Introduction

Contrast agents are administered during magnetic resonance imaging (MRI) to improve the visualization of anatomical and pathological features in the human body [1, 2]. The standard-of-care recommends a dose of 0.1 mmol Gd/kg for most gadolinium-based contrast agents (GBCAs), and of 0.05 Gd/kg for new generation agents like gadopiclenol [3].

In recent years, AI-based solutions have been proposed in the context of contrast-enhanced MRI, addressing three related but distinct tasks: contrast amplification, dose reduction and synthetic dose prediction (or virtual contrast). Given a clinically standard Gd dose, contrast amplification aims to generate post-contrast images characterized by an increased enhancement compared to the original acquisition [4–6]. Dose reduction approaches instead focus on restoring standard-level contrast enhancement after injecting a reduced dose of contrast agent [7–11]. Finally, synthetic dose prediction seeks to reproduce a standard post contrast image without any injection of contrast agent, relying on the hypothesis that contrast-related information can be inferred from other MRI sequences, such as T2-weighted or diffusion-weighted imaging [12–17]. This work specifically focuses on the contrast amplification paradigm.

The dose of contrast agent is typically determined based on considerations of both efficacy and safety. However, in certain cases, the use of higher-than-recommended doses may be beneficial — for example — to improve the detection of small, low-enhancing lesions [18–20].

Within this framework, AI contrast amplification approaches can be viewed as a solution to enhance lesion visibility without increasing gadolinium exposure.

Among the AI-based methods proposed to amplify the effects of GBCAs in brain MRI scans, one approach [4] uses T1-weighted pre-contrast and standard post-contrast images as input and is based on a convolutional neural network that selectively enhances regions of the brain that exhibit contrast uptake at standard dose demonstrating quantitative contrast amplification and improved lesion conspicuity, e.g. in small multiple sclerosis plaques and metastatic lesions.

The network was trained and validated using imaging data from adult patients. Extending this approach to pediatric populations is challenging due to the limited availability of high-quality pediatric MRI datasets. This scarcity of data hinders the development of robust, generalizable models tailored to the specific anatomical and pathological characteristics of pediatric patients. Recent studies have explored the application of AI methods in pediatric imaging [21, 22]; however, the amount of training data remains limited, and further efforts are needed to expand and validate these approaches in this specific population.

In this study, we hypothesize that the AI network, trained on adult brain MRI data, (i) effectively increases contrast within contrast-enhancing lesions and (ii) preserves anatomical fidelity across distinct patient populations, specifically pediatric patients. To test this hypothesis, we performed a quantitative analysis and a multi-reader clinical performance assessment of the algorithm applied to pediatric patients (0–17 years) diagnosed with various brain tumors. This study is designed as a feasibility and cross-domain validation rather than a population-level performance study, acknowledging the limited sample size and potential constraints on generalizability.

## Materials and methods

### Dataset

Multiparametric MR scans of 22 pediatric patients (13 male, 9 female), Ethics Committee of Liguria, Genoa, Italy (registration number 006/2019), were selected based on: (i) availability of axial T1W gradient echo or spin echo image before (PRE) and after GBCA administration (standard dose (SD), 0.1 mmol Gd/kg), acquired with identical parameters (echo time (TE), repetition time (TR), geometrical orientation, image resolution); (ii) absence of motion artefacts; (iii) successful co-registration between SD and PRE (visually verified).

### Preprocessing

Data were anonymized using Anonymizer Pro (NeoLogica srl, Italy) and linearly rescaled using RescaleSlope (0028,1053) and RescaleIntercept (0028,1052) DICOM tags. To be used as input for the deep learning model, imaging sequences were then co-registered with an affine registration procedure (SimpleElastix [23]); the default affine parameter map was adopted, using advanced Mattes mutual information as similarity metric, optimized via adaptive stochastic gradient descent, with linear interpolation and a multi-resolution image pyramid.

Finally, consistent with the pre-processing of the training data, scans were linearly normalized in the [0,1] range by dividing each pair of images of the same patient by the maximum value of the SD image. The SD was chosen as the reference because it is expected to exhibit the maximum intensity value.

### Deep learning method

The deep learning algorithm developed by Fringuello Mingo et al. [4] was applied to predict artificial double-dose images (A-DD) using PRE and SD images as input. The AI model consists of a U-Net-like encoder-decoder convolutional neural network with 3 layers per branch and skip connections between corresponding levels of the encoder and decoder structures (see Supplementary information for additional details). The model was trained on about 2000 Cerebral Nervous System (CNS) contrast enhanced MRI procedures in adult subjects with PRE and SD contrast scans; the amplification algorithm was firstly validated through an ad hoc designed preclinical study, which involved forty-eight Wistar rats injected with standard and double dose of contrast agent, in order to have the real Double Dose (DD) images as Ground Truth; then, the algorithm was tested on adult patients and clinically evaluated in two clinical studies, which compared the A-DD* with the SD images [4].

### Post-processing

The predicted images with amplified contrast dose underwent an additional post-processing step, based on the High Dynamic Range (HDR) algorithm, to increase dynamic range, texture realism and local intrinsic contrast, especially in non-enhancing brain parenchyma regions.

The HDR algorithm was identically applied across all subjects and consisted of computing a voxel-wise weighted linear combination of the amplified image with the two T1W input images. The weighting coefficients are modulated across the images so that: (i) in non-enhancing regions of the image, the weights of PRE and SD images are higher, and (ii) in enhancing regions the amplified image gives the highest contribution to pixel intensity. The resulting image (A-DD*) is characterized by a further increase in image quality and local contrast in healthy or non-enhancing tissue. The algorithm has been clinically validated previously [24]. Further details are recalled in the Supplementary information section.

### Quantitative analysis

SD scans were compared with A-DD* images by evaluating the lesion-to-brain ratio (LBR), the contrast-to-noise ratio (CNR) and the contrast-enhancement percentage (CEP):

$$\begin{array}{ccc}LBR=\frac{{SI}_{lesion}}{{SI}_{brain}}& CNR=\frac{{SI}_{lesion}-{SI}_{brain}}{{StDev}_{brain}}& CEP=100*\frac{{SI}_{lesion}-{SI}_{lesion}^{pre}}{{SI}_{lesion}^{pre}}\end{array}$$

where SI and StDev denote the average signal intensity (SI) and its Standard Deviation (StDev) sampled from Regions Of Interest (ROI) placed in pathological (lesion) and normal appearing (brain) tissues.

ROIs were placed by an expert neuroradiologist (**, 20 years of experience) in the largest homogeneous enhancing area of the tumor lesion and in the contralateral cerebral parenchyma (avoiding large blood vessels); the ROI placement protocol was predefined in agreement with the clinician and, while no formal intra- or inter-rater variability assessment was performed, its robustness was assessed during a preliminary acclimatization period.

**Fig. 1:**
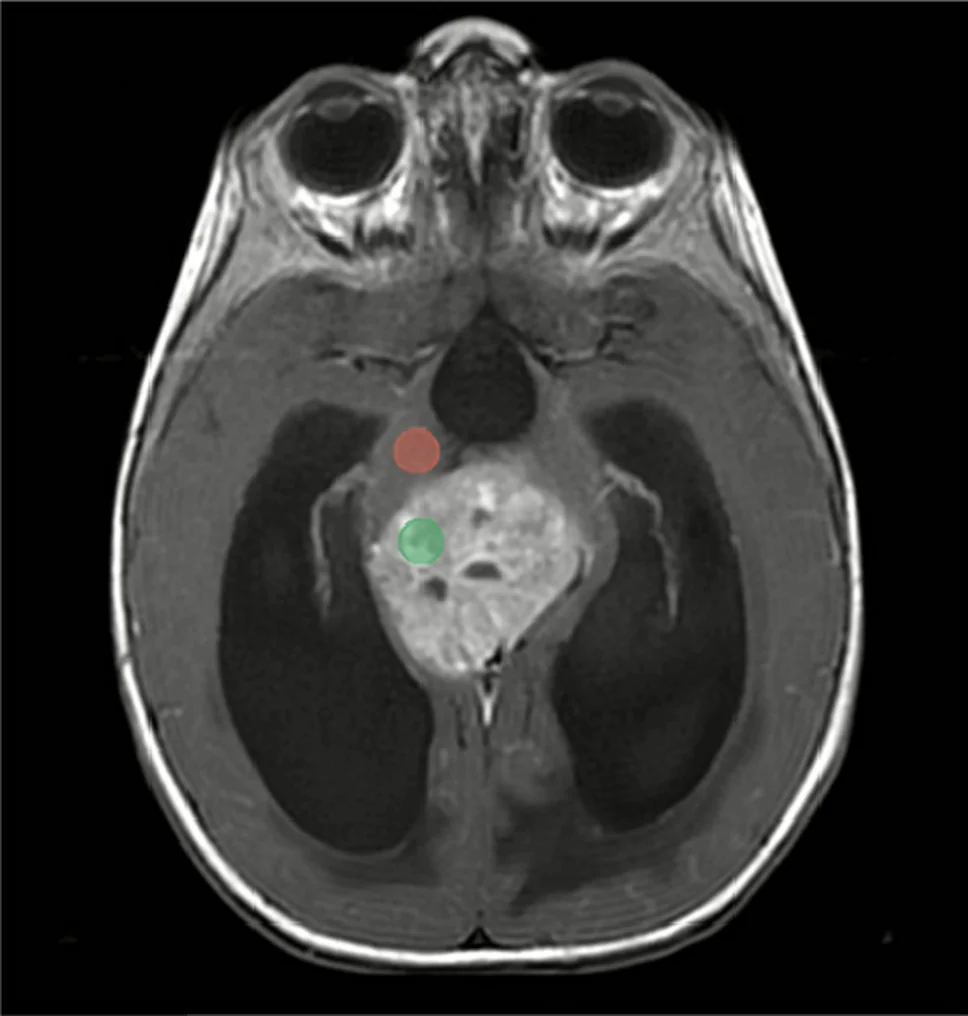
ROI positioning (green Lesion ROI, red parenchyma ROI) for patient t

A representative example of ROIs positioning is provided in Fig. 1. Data normality was assessed using the Shapiro–Wilk test. Statistical differences were compared using a two-tailed paired t-test. The effect size was also calculated, using the Cohen’s difference metric. Additionally, percentage variations of the three parameters (LBR, CNR, CEP) were calculated.

The quantitative analysis was performed on a total of 19 cases (three subjects were excluded as the corresponding brain lesions did not show any contrast enhancement) and on subsets of cases defined according to age (0–2 y/o n = 10, 2–17 y/o n = 9). Subjects aged 0–2 years are expected to exhibit the most pronounced anatomical differences relative to the adult images used to train the network.

### Reading study

SD and A-DD* images were qualitatively evaluated by three neuroradiologists (**,**,**, 20,1 and 5 years of experience), blinded to the image source and free to adjust windowing for each image at will. Readers independently graded Image Quality, Presence of imaging artifacts, Lesion Border Delineation, Visualization of Lesion Internal Morphology, Degree of Contrast Enhancement and Vessel conspicuity using a 4-point Likert scale (Table 1). Statistical differences were compared using a 2-tailed t-test. Furthermore, the readers were asked to express an overall preference between SD and A-DD* for each patient.

**Table 1:** Qualitative evaluation criteria

Criteria	1 (Poor)	2 (Fair)	3 (Good)	4 (Excellent)
Image quality	Blurry, lacks detail	Some clarity, low sharpness	Clear with good detail	Very clear, sharp, and detailed
Imaging artifacts	Many artifacts, hard to interpret	Some artifacts, partially affect quality	Few artifacts, minimal impact	No artifacts
Lesion border delineation	Not visible/poorly defined	Partially visible, unclear	Mostly visible, fairly defined	Clearly visible and well-defined
Lesion internal morphology	Internal structure not visible	Partially visible, poorly defined	Mostly visible, fairly defined	Clearly visible and well-defined
Contrast enhancement	No enhancement	Minimal enhancement	Noticeable enhancement	Strong and clear enhancement
Vessel conspicuity	Overly visible vessels, risk of misdiagnosis	Moderate visibility, some misdiagnosis risk	Adequate visibility, low misdiagnosis risk	Optimal visibility

### Anatomical similarity analysis

A-DD* images were quantitatively compared to SD images to evaluate anatomical similarity. Firstly, the Structural Similarity Index Measure (SSIM) [25], a common similarity metric, was calculated to evaluate the anatomical correspondence between A-DD* and SD images. SSIM values close to 1 suggest high structural similarity between the compared images, while values approaching 0 indicate substantial structural differences. Furthermore, the SD and A-DD* images for each subject were co-registered to the corresponding pre-contrast image using a nonlinear registration algorithm (FSL/FNIRT [26]), and the resulting warp fields were computed. Following skull-stripping with SynthSeg [27] to remove most non-brain tissue and background, the log-Jacobian range was calculated to quantitatively assess anatomical differences. Specifically, the log-Jacobian range measures spatial distortion by quantifying local volume changes introduced during registration: values greater than zero indicate expansion, while values less than zero indicate contraction [28, 29], supporting the preservation of anatomical structure. The log-Jacobian ranges obtained from the SD and A-DD* images were then statistically compared using a paired t-test. This metric complements SSIM: while SSIM evaluates intensity and structural similarity, the log-Jacobian specifically captures geometric consistency. The combination of these two measures provides a more comprehensive assessment of anatomical fidelity.

## Results

A total of 22 patients (0–17 y/o; 9 Females, 13 Males), were considered eligible for the Reading Study. For the quantitative assessment, only patients characterized by an enhancing lesion were included.

All subjects, including those with non-enhanced lesions were included in the qualitative assessment and anatomical similarity analysis.

**Fig. 2:**
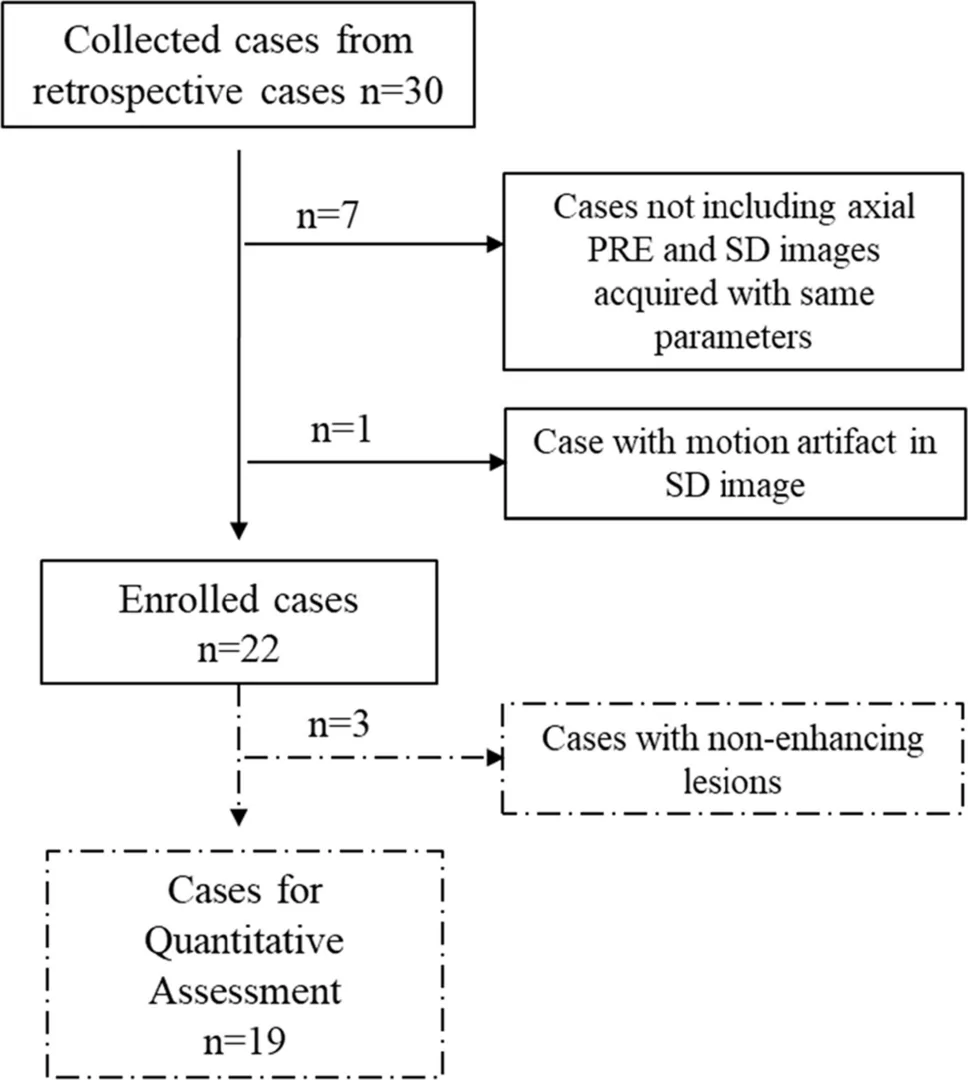
Flowchart of the study for patients’ cohort selection

The selection process is shown in Fig. 2.

Images were acquired using Philips 1.5 T or 3 T scanners (Intera Achieva, Ingenia CX, Philips, Best, the Netherlands), following the European Society for Pediatric Oncology (SIOPE) MRI guidelines for imaging patients with CNS tumors [30]. Table 2 details age, gender and pathology, including Tumor Type and World Health Organization (WHO) Grade [31], which is primarily based on the histological characteristics and molecular markers of the tumor, and represents a global reference for diagnosis, research, cancer registers.

**Table 2:** Demographic table of pediatric population (Patient ID, tumor subtype and age) during the imaging session. NA: not applicable

Patient ID	Age (y/o)	Sex	Tumor subtype	Who Grade
a	16	M	Diffuse midline glioma, H3K27-altered	4
b	7	F	Diffuse midline glioma, H3K27-altered	4
c	3	M	Pilocytic Astrocytoma	1
d	7	F	Pilocytic Astrocytoma	1
e	17	F	Diffuse midline glioma, H3K27-altered	4
f	11	F	Diffuse midline glioma, H3K27-altered	4
g	9	M	Pilocytic Astrocytoma	1
h	13	F	Diffuse midline glioma, H3K27-altered	4
i	11	F	Ganglioglioma	1
j	4	M	Diffuse pediatric-type high-grade glioma, H3-wildtype, and IDH-wildtype	4
k	9	M	Diffuse pediatric-type high-grade glioma, H3-wildtype and IDH-wildtype	4
l	15	F	Diffuse midline glioma, H3K27-altered	4
m	7 months	M	Posterior fossa ependymoma	2
n	2	M	Posterior fossa ependymoma	3
o	1	F	Pilocytic Astrocytoma	1
p	1	F	Retinoblastoma	NA
q	1	M	Pineoblastoma	4
r	1	F	Medulloblastoma, SHH-activated and TP53-wildtype	4
s	1	M	Pilocytic Astrocytoma	1
t	6 months	M	Pineoblastoma	4
u	2	M	Posterior fossa ependymoma	3
v	2	M	Medulloblastoma, non-WNT/non-SHH	4

**Fig. 3:**
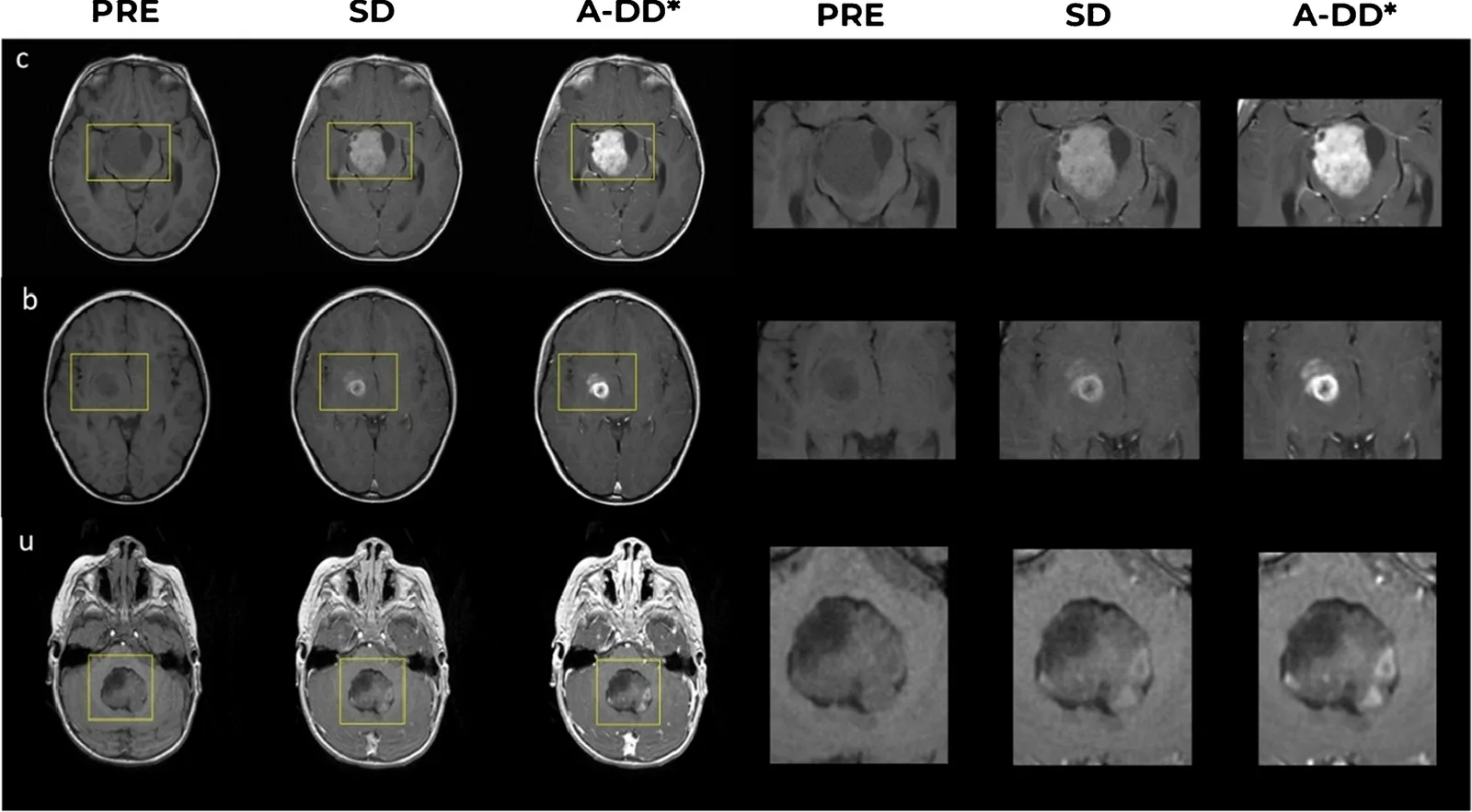
PRE, SD and A-DD* images of 3 representative patients. For each patient, the whole brain image and the lesion zoom are reported. Letters in the top left corner represent Patients ID, as reported in Table 1. The yellow squares indicate the zoomed area

Figure 3 shows PRE, SD and A-DD* images for three subjects with varying contrast enhancement patterns. The A-DD* image demonstrates visibly enhanced contrast relative to the SD image, without any distortion of brain anatomy or degradation of image quality, as illustrated in the zoomed-in regions. Patient ‘c’ represents a tumor with complex internal morphology; while contrast is visibly enhanced, the tumor’s structural features are preserved. In case ‘b’ the central necrotic area of the tumor remains unaltered, while the ventral contrast-enhancing components of the lesion become more clearly visible on A-DD*. Likewise, in patient ‘u’, two small, faint regions of contrast enhancement that are not particularly prominent in the SD image appear more distinctly on A-DD* images.

**Fig. 4:**
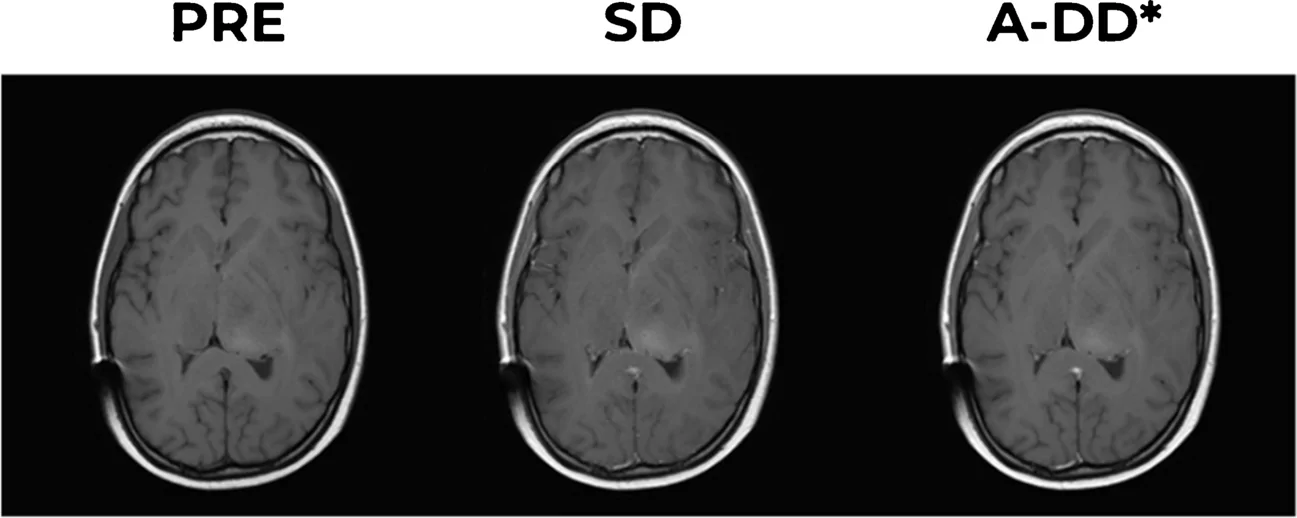
PRE, SD and A-DD* scans of patient ‘e’, which doesn't show any contrast artifacts after the contrast amplification algorithm

Figure 4 presents a bilateral thalamic glioma which exhibits a mild spontaneous T1 hyperintensity of the left thalamus (visible on the PRE image), but does not demonstrate increased enhancement following contrast injection.

Quantitative analysis confirms increased signal intensity in contrast-enhancing regions, both relative to surrounding tissue and to pre-contrast images. Indeed, LBR, CNR and CEP values are significantly higher in the A-DD* images compared to the corresponding SD acquisitions (Cohen's d CEP = 0.859, Cohen's d CNR = 1.291, Cohen's d LBR = 1.342).

**Fig. 5:**
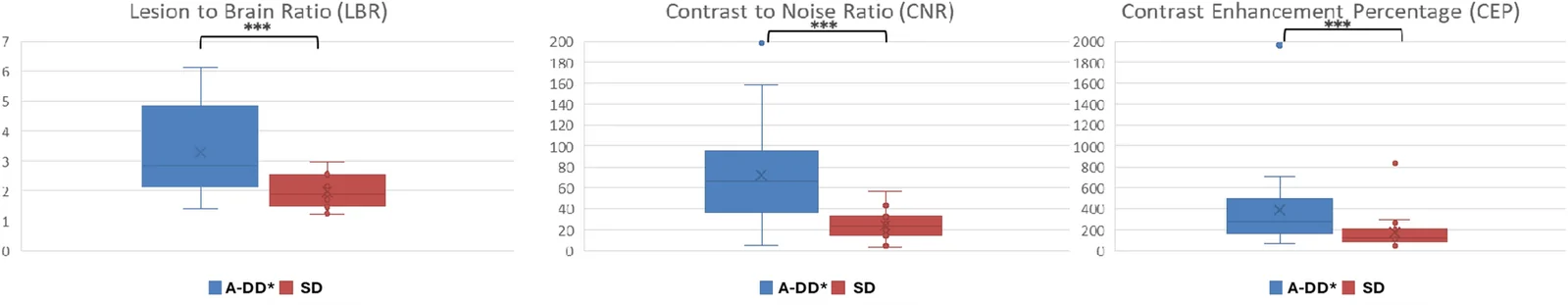
Quantitative comparisons between A-DD* and SD images for LBR, CNR, CEP. *p < 0.05, **p < 0.01, ***p < 0.001

Averaging over the n = 19 cases analyzed, percentage increases of 61.9%, 186.5% and 110.4% were observed, respectively. The box plots of the values for both SD and A-DD* images are shown in Fig. 5, in order to show the overall distribution of metrics across the population; results obtained for each age-based subset are reported in the Supplementary information, particularly in the 0–2 y/o subgroup the percentage increases were 47.9%, 153.9% and 98.5%, confirming the hypothesis of the robustness of the network also in the presence of significant anatomical differences.

Qualitative assessment was independently performed by the three readers on the whole dataset (n = 22). The A-DD* images were considered equivalent to the SD images across all criteria, with no statistically significant differences observed (Table 3), except for Image Quality which was perceived higher in the A-DD* for the Reader 2. Considering the average between readers, Image Quality was graded 3.75 for SD images whereas 3.86 for A-DD* images (*p-*value = 0.05); Artifact Presence 3.80 for SD images and 3.80 for A-DD* (*p-*value = 1); Lesion Border Delineation 3.93 for SD and 3.95 for A-DD* (*p-*value = 0.65); Lesion Internal Morphology 3.95 for both SD and A-DD* (*p-*value = 1); Degree of Contrast Enhancement 3.92 for SD image and 3.95 for A-DD* (*p-*value = 0.48) and finally Vessel conspicuity was graded 3.95 for both images (*p-*value = 1). However, when asked to express an Overall Preference, the A-DD* image was favored in the majority of cases: 12 out of 22 cases (54.5%) by Reader 1, 18 out of 22 cases by Reader 2 (81.8%) and 13 out of 22 cases for Reader 3 (59.1%). Cohen’s kappa for the overall preference expression resulted in 0.646 for R1-R3 accordance, while 0.240 for R1-R2 and R2-R3 evaluations.

**Table 3:** Qualitative assessment results per reader and criterion on SD and A-DD images. Statistical differences between SD and A-DD* are reported for each reader and for the average score across readers

	Image quality			Artifact presence			Lesion Border delineation		
	SD	A-DD*	SD vs A-DD*	SD	A-DD*	SD vs A-DD*	SD	A-DD*	SD vs A-DD*
Reader average	3.7(0.43)	3.86(035)	*p = *0.05	3.80(0.40)	3.80(0.40)	*p = *1	3.93(0.24)	9.35(0.21)	*p = *0.66
Reader 1	3.77(0.43)	3.77(0.43)	*p = *1	3.82(0.39)	3.77(0.43)	*p = *0.72	4(0)	4(0)	
Reader 2	3.73(0.46)	3.95(0.21)	p < 0.0.5*	3.86(0.35)	3.86(0.35)	*p = *1	3.86	3.86(0.35)	*p = *0.64
Reader 3	3.77(0.43)	3.86(0.21)	*p = *0.33	3.73(0.46)	3.77(0.43)	*p = *0.58	3.96(0.21)	4(0)	*p = *0.33
	SD	A-DD*	SD vs A-DD*	SD	A-DD*	SD vs A-DD*	SD	A-DD*	SD vs A-DD*
Reader average	3.75(0.43)	3.95(0.21)	*p = *1	3.92(0.26)	3.95(0.21)	*p = *0.48	3.95(0.21)	3.95(0.21)	*p = *1
Reader 1	3.77(0.43)	3.95(0.21)	*p = *0.32	3.91(0.29)	4(0)	*p = *0.15	4(0)	4(0)	
Reader 2	3.73(0.46)	3.91(0.29)	*p = *0.65	3.95(0.21)	3.91(0.29)	*p = *0.56	3.86(0.35)	3.91(0.29)	*p = *0.65
Reader 3	3.77(0.43)	4(0)		3.91(0.29)	3.96(0.21)	*p = *0.58	4(0.35)	3.96(0.21)	*p = *0.33

**Fig. 6:**
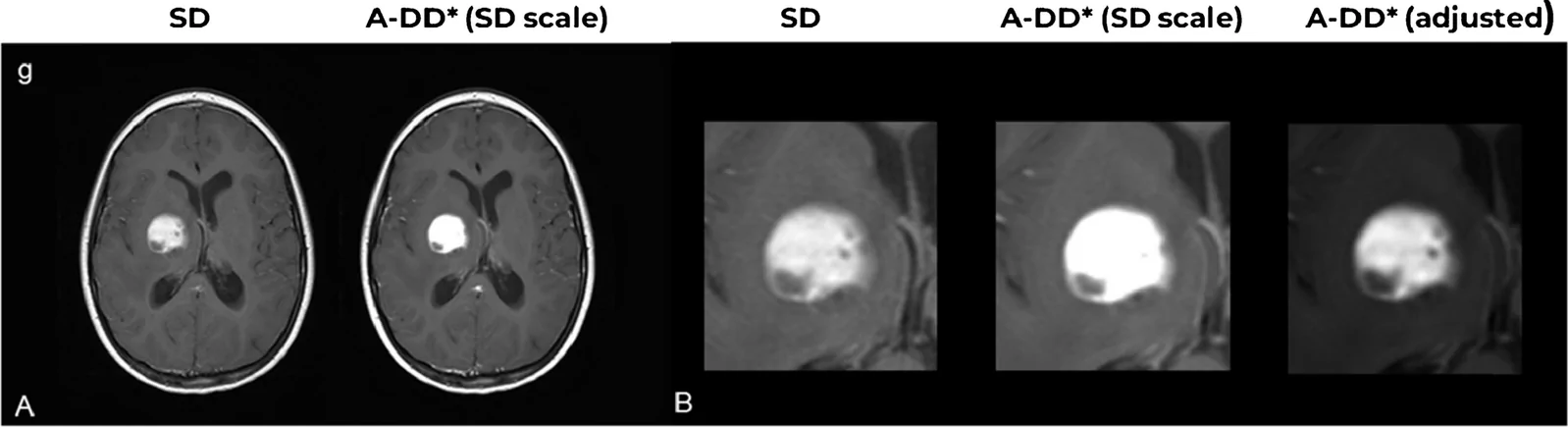
Representative case of patient g, in which all readers preferred SD to A-DD* (Panel **A**). Lesion zoom with Intensity scale adjustment results (Panel **B**). From left to right: SD with optimized gray-scale, A-DD* with the same gray-scale as A-DD*, A-DD* with optimized gray-scale

Figure 6 shows an example in which all readers preferred the SD image to the A-DD*. Indeed, in a few cases, the algorithm consistently extends the signal intensity range of the A-DD* image, resulting in apparent signal saturation within the lesion (Fig. 6A). In this subject, two small medial cystic components of the lesion visible in the SD image are not easily discernible in the A-DD* image when displayed using the same intensity scale. However, as shown in Fig. 6B, the internal morphology of the tumor is preserved in the A-DD* image and becomes clearly visible upon appropriate adjustment of the grayscale window.

The analysis of the anatomical similarity demonstrated the anatomical fidelity of the A-DD* images with respect to the correspondent SD images, both in terms of SSIM and co-registration metrics. The SSIM values between A-DD* and SD images ranged from 0.949 to 0.995 (mean = 0.98, StDev = 0.012), assessing almost perfect similarity between the amplified and standard dose images.

**Fig. 7:**
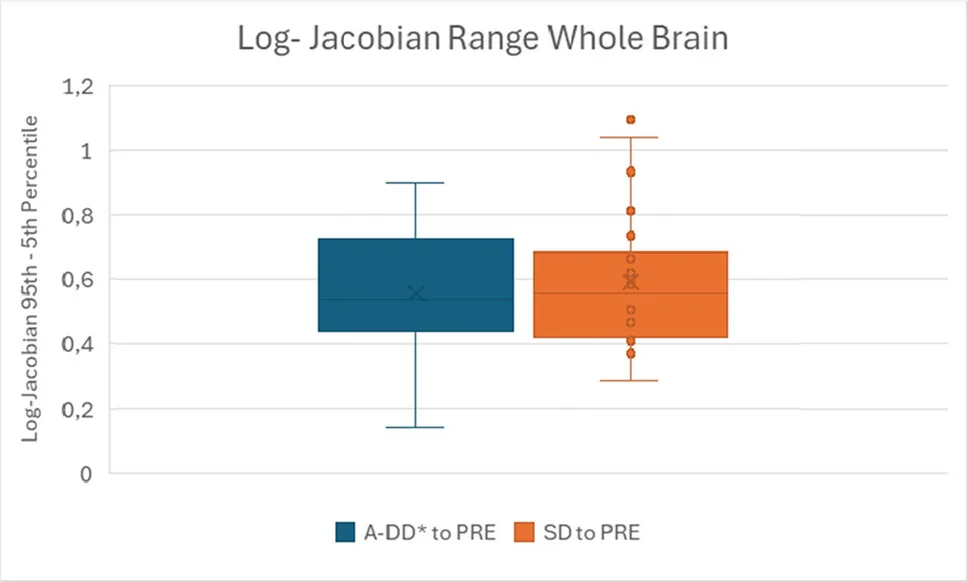
Box Plots of Log-Jacobian Range values for SD to PRE and A-DD* to PRE for whole brain and for the lesion-specific region

Moreover, no statistically significant differences (Fig. 7) were observed for range of Log-Jacobian Range (RANGE SD mean = 0.592, RANGE A-DD* mean = 0.558, paired t-test *p-*value = 0.556), suggesting that nonlinear deformations fields are not affected by contrast amplification algorithm; this indicates that the network, although trained on adult brain images, does not introduce any detectable structural deformation while amplifying contrast of MR images at earlier brain developmental stages.

## Discussion

In pediatric brain MRI, obtaining sufficiently large and representative training datasets for AI models remains a significant challenge [32]. Moreover, particularly in children aged 0–2, the brain undergoes transitional changes characterized by pronounced morphometric alterations and variability in intrinsic contrast due to ongoing myelination [33]. These challenges hinder the development of robust AI-enhanced diagnostic imaging tools for the pediatric brain [34].

We hypothesize that an AI tool for brain imaging developed and validated in adult populations [4] may also be applicable to pediatric patients, despite anatomical and developmental differences. This assumption is based on the design of the AI network, which functions by amplifying exogenous contrast, such as that introduced by contrast agents, without altering the underlying anatomical structures or the intrinsic tissue contrast present in the original MRI scans. Because the algorithm does not modify age-dependent features or introduce artificial structural changes, we propose that its effect is independent of developmental stage, allowing it to enhance image contrast in both adult and pediatric brain MRIs without compromising diagnostic integrity. Given the limited patient cohort, these findings should be interpreted with caution, particularly regarding their generalizability to broader clinical populations, and considered as a preliminary step toward further evaluation in larger pediatric cohorts.

To evaluate the performance of the AI-based contrast amplification tool, a quantitative analysis was conducted first. The observed increases in contrast enhancement parameters, specifically contrast enhancement percentage (CEP), contrast-to-noise ratio (CNR), and lesion-to-background ratio (LBR), are attributable to contrast amplification within contrast-enhancing lesion areas, while maintaining stable signal intensity and contrast in the surrounding brain parenchyma. This improvement in the quantitative metrics reflects that the algorithm’s effect goes beyond a simple “windowing” adjustment; indeed, the enhancement amplification introduces a genuine change in contrast metrics that would not be measurable through standard windowing alone, which only alters the visual appearance of the image. Importantly, the network preserves anatomical structures and local intrinsic contrast, ensuring the integrity of the original image content. The value of the tool is evident in the cases presented in Fig. 3 of the Results section. For example, despite the complex internal morphology, the tumor of patient ‘c’ acquires evident increased enhancing in the A-DD* image, suggesting that amplifying contrast enhancement may facilitate delineation of the tumor burden. Different application but same value is confirmed for patient “u”, where two regions with low intrinsic contrast could be more distinctly visible on A-DD* images.

Moreover, another representative case has been shown in Fig. 4: a patient characterized by a spontaneous mild contrast visible on the T1 pre-contrast did not show any augmented enhancement: this highlights that the amplification algorithm does not act on physiological hyperintensities but selectively operates on regions characterized by contrast uptake.

Qualitative assessment by independent readers in a single-blind study design further supports these findings, with A-DD* images receiving scores close to “excellent”, and image quality considered superior to SD scans by Reader 2. No statistically significant differences were observed in the remaining qualitative scores. This result should be interpreted in light of a ceiling effect of the Likert scale; indeed, SD images were consistently rated very highly (mean score 3.75 on a 4-point scale), leaving limited dynamic range for detecting incremental improvements. As a result, the Likert scale may not be sufficiently sensitive to capture subtle but clinically relevant differences between two already high-quality image sets.

For this reason, the qualitative results are more appropriately interpreted as demonstrating non-inferiority, confirming that A-DD* images preserve image quality, do not introduce artifacts, and do not generate spurious contrast enhancement.

The most evident benefit of A-DD* images emerges from complementary findings including quantitative improvement in all contrast related metrics, consistent with an overall reader’s preference. Indeed, all readers expressed an overall preference for the contrast-amplified images over the SD counterparts, indicating that the improvement is evident both quantitatively and qualitatively. Even if all the readers expressed a preference for the A-DD*, the most significant result is reached for Reader 2, the least experienced neuroradiologist, who preferred the A-DD* for over the 80% of cases. Inter-reader variability is a known and expected factor in radiological image interpretation studies [35, 36]. Readers 1 and 3 showed substantial agreement (κ = 0.646), whereas the agreement with the younger reader was only slight (κ = 0.240). This observed variability may reflect differences in reader experience and familiarity with contrast-enhanced imaging, particularly in the context of novel image processing techniques. Moreover, Reader 2 perceived a statistically significant improvement in image quality with A-DD* compared to SD. These findings suggest that A-DD* could be considered more readable and with improved clarity, indicating that the contrast amplification algorithm could constitute a valuable tool for radiologists at the beginning of their clinical careers, or even for experienced professionals working under high workload conditions, such as at the end of a long day with many cases reviewed; conversely, more experienced radiologists might require some acclimatization to adjust to the contrast amplified scans, as their diagnostic routine is more established.

Figure 6 illustrates one of the few cases in which the SD was preferred to the A-DD* by all three readers, due to apparent signal saturation within the enhancing lesion. As described in the Results section, this effect is readily reversible through an appropriate adjustment of the grayscale window. This observation does not reflect a failure of the algorithm; however, it highlights the need for clinicians to become familiar with the new image. The tool correctly preserves tumor heterogeneity and anatomical information; thus, with minimal training clinicians can confidently interpret A-DD* images and fully benefit from the enhanced information they provide.

We demonstrated that the AI algorithm selectively works on contrast amplification without introducing any anatomical distortion in the A-DD* images. We evaluated it by directly measuring the similarity between A-DD* and SD images through the SSIM metric, and then by confirming that no statistically significant differences emerged when comparing the deformations, in terms of Log-Jacobian Range, resulting from co-registering A-DD* and SD to the corresponding PRE contrast image.

Finally, we note that similar trends were observed in the 0–2 years subgroup (see Supplementary information). This developmental stage is characterized by rapid changes in the central nervous system, potentially introducing greater inter-individual variability in both morphological and physiological features. Although this subgroup analysis is limited by the small sample size and should be considered exploratory, the consistency of the observed effects provides preliminary support for the robustness of the approach across different developmental stages.

This study has several limitations that warrant consideration. The most significant is the small sample size, which reflects the inherent difficulty of collecting imaging data from pediatric patients with brain tumors, a population that is both clinically vulnerable and statistically rare. However, it is important to emphasize that the pediatric population has been used exclusively as test set for the algorithm, and not for training purposes; the test set of this study has a sample size comparable with several dose reduction or contrast amplification studies which include clinical evaluation, which often include a few tens of patients[6, 10, 11]. For instance, Haase et al. [6] included 30 patients in the test set of their study on contrast amplification for brain metastasis identification, as well as in their study on dose reduction algorithm. Furthermore, it is crucial to highlight that our study involves pediatric patients, who represent a clinically more delicate and complex population compared to adults.

Additionally, the ROI-based quantitative analysis relies on manual ROI placement and is therefore inherently susceptible to operator-dependent variability. Because no formal intra- or inter-observer reproducibility analysis was performed, the impact of ROI selection on the quantitative metrics could not be directly assessed.

Additionally, the study lacked access to true double-dose contrast-enhanced images, which restricts the ability to directly compare the AI-enhanced images to clinically acquired high-dose references. It is worth noting, however, that the same network has been evaluated in preclinical models of brain tumors, where true double-dose acquisitions were available, and demonstrated excellent performance in predicting the exogenous contrast enhancement associated with higher doses of contrast agent.

To overcome these limitations, several next steps are envisioned. Future work will focus on validating the proposed algorithm in larger and more heterogeneous cohorts, ideally including multi-center data to improve generalizability. In addition, prospective studies would represent the optimal setting to assess the clinical impact of AI-based contrast enhancement in routine practice. Such investigations will be essential to move beyond technical validation and to determine the potential added value of this approach in clinically relevant tasks, particularly in the pediatric population. Finally, the aim of our study is to assess the technical effectiveness of the contrast enhancement algorithm on a cohort of pediatric patients. However, the scientific community is currently working on proving the clinical value of AI contrast amplification across different applications, such as improved detection of micro-metastases, more precise lesion margin delineation, and earlier identification of recurrence or residual disease. Notably, existing literature already provides preliminary evidence that similar algorithms may contribute to an improvement of clinical outcomes, in particular in the context of metastasis identification, However, this is still a highly active and evolving area of research [23].

In conclusion, the findings of this study indicate that a deep learning–based contrast amplification model trained on adult data can generalize to pediatric brain MRI, enhancing the effects of GBCAs while preserving anatomical fidelity. This is supported by both quantitative metrics and qualitative evaluation, as well as by anatomical similarity analyses demonstrating the absence of structural distortion. These results suggest that this approach may facilitate more detailed lesion characterization in pediatric imaging. However, potential benefits in clinical workflow and diagnostic decision-making remain to be established in prospective studies. Within this context, artificial contrast amplification may represent a promising supportive tool in pediatric brain tumor imaging, where accurate and high-quality imaging is essential for diagnosis, treatment planning, and follow-up.

## Supplementary Information

Below is the link to the electronic supplementary material.

Supplementary file1 (DOCX 63 KB)

## Data Availability

Data are not public available. They are part of a retrospective observational study approved by the Regional Ethics Committee of Liguria, Genoa, Italy (registration number 006/2019). Informed consent.

## Abbreviations

AI: Artificial Intelligence
A-DD: Artificial Double-Dose (AI predicted images)
A-DD*: Post-processed A-DD images using HDR algorithm
CEP: Contrast Enhancement Percentage
CNR: Contrast-to-Noise Ratio
CNS: Central Nervous System
DD: Double dose
DL: Deep Learning
DICOM: Digital Imaging and Communications in Medicine
FSL: FMRIB Software Library (toolbox for brain images analysis)
FNIRT: FMRIB's Non-linear Image Registration Tool
GBCA: Gadolinium-Based Contrast Agent
HDR: High Dynamic Range
LBR: Lesion-to-Background Ratio
MRI: Magnetic Resonance Imaging
PRE: Pre-contrast image
ROI: Region of Interest
SD: Standard Dose (post-contrast image acquired with standard contrast dose)
SI: Signal Intensity
SSIM: Structural Similarity Index Measure
SIOPE: European Society for Pediatric Oncology
TE: Echo Time
TR: Repetition Time
T1w: T1-weighted
WHO: World Health Organization
y/o: Years Old
