# Supplementary material for: Deep learning for contrast-enhanced MRI in pediatric brain imaging
Source: Neuroradiology. 2026 Jul 4;68(9):3059–69. doi: 10.1007/s00234-026-04073-x (PMC13680331; doi:10.1007/s00234-026-04073-x)
Supplement: Supplementary file 1 [file 234_2026_4073_MOESM1_ESM.docx]

SUPPLEMENTARY INFORMATION

***TRAINING PARAMETERS***

The AI model used in this study was originally trained on adult data and applied in pediatric patients. Additional details on the network architecture and training parameters are provided in Table 4.

| **NEURAL NETWORKS PARAMETERS** | |
| --- | --- |
| **Input data** | **Input channels:** PRE and SD images.  **Data augmentation trasformations:** Rotation+Flip |
| **Training/validation split** | 70% training set  20% validation set  10% test set |
| **NN Architecture** | U-net with residual and skip connections |
| **Training parameters** | **Loss function:**  3-component composite loss: mean absolute error (MAE), MAE in the Fourier transform space, and perceptual loss (V-GG network) with three weight coefficients.  **N. Epochs:** 250  **Learning rate:** 0.01  **Decay:** 0.001  **Optimizer:** Stocastic gradient descend (ADAM) |

Table 4 Detailed specifications of the network architecture and training settings for the AI model trained on adult data

***HDR algorithm implementation***

An HDR algorithm inspired by photography was implemented. In photographic applications, HDR relies on exposure fusion and tone mapping to retain detail across the full luminance range. Here, the objective is to preserve details that are lost in the native A-DD image in the areas with low enhancement due to exogenous contrast In this implementation there is a ‘bright’ image, the PRE contrast, a ‘normal’ image, the SD and a ‘dark’ image, the A-DD. Weights are computed based on grayscale copies of the normal and dark image that are used for weighting the normal and dark images. An inverted copy of the bright image was used for weighting the bright one. A voxel value in the resulting image was determined by the weighted sum of the three acquisitions, where the weights are expressed as function of the aforementioned copies of the images. Using the dark and normal images as their own weighting image gives them a lower weight where the scene is dark (areas with low exogenous contrast), and more weight where the scene is brightest (areas with high exogenous contrast). Conversely, the weighting applied to the bright image (i.e., the inverted bright image) emphasizes regions where the scene is darker. This choice is driven by a phenomenological observation indicating that such a combination enhances the distinction between white and gray matter—an important contrast that is often reduced in post-contrast images and even more so in artificially amplified ones.

Finally, the weighting coefficients (wn, wd, wb) are set empirically to adjust each image's contribution to the final HDR image. The goal was to enhance visibility of anatomical structures in areas unaffected by exogenous contrast.

The above reported description can be translated into the following equations:

$${A-DD}^{*}={weight}_{bright}*bright+{weigh}_{dark}*dark+ {weight}_{normal}*normal$$

where:

${weight}_{bright}=w_{b}*\frac{{bright}_{inverted}}{norm}$,

${weight}_{dark}=w_{d}*\frac{dark}{norm}$,

${weight}_{normal}=w_{n}*\frac{normal}{norm}$,

$$norm=w_{b}*bright_{inverted}+w_{d} *dark+w_{n}*normal$$

In the current study the following values for the weight coefficients were used: wb =1; wd =3; wn =3.

These values were selected empirically based on visual inspection of the images while varying the parameters. Although a formal sensitivity analysis was not conducted, a qualitative exploration of the parameter space indicated that this combination provided a favorable trade-off, restoring the visibility of gray levels in the parenchymal regions while maintaining contrast amplification within the enhanced lesion areas.

***Subgroup analysis***

A sub-group analysis according to the age of the subjects was performed with the aim of assessing the performance of the contrast amplification method in children aged between 0 and 2 years (Figure 8 ), known to present more variable and distinct morphometric features compared to older patients, and in children aged 3-7 y/o (Figure 9).

Figure 8. Quantitative Comparisons between A-DD* and SD images for LBR, CNR, CEP on pediatric patients with age between 0-2 y/o. *p<0.05, **p<0.01, ***p<0.001

Figure 9. Quantitative Comparisons between A-DD* and SD images for LBR, CNR, CEP on pediatric patients with age between 3-17 y/o. *p<0.05, **p<0.01, ***p<0.001

Results confirm a significant increase in LBR, CNR and CEP of A-DD* images compared to SD acquisitions for both age groups. This evidence further confirms the strength of the contrast amplification method over anatomical differences.
